# Brain areas interconnected to ventral pathway circuits are independently able to induce enhancement in object recognition memory and cause reversal in object recognition memory deficit

**DOI:** 10.1111/cns.14727

**Published:** 2024-04-21

**Authors:** Mariam Masmudi‐Martín, Irene Navarro‐Lobato, Manuel F. López‐Aranda, María E. Quiros‐Ortega, Marta Carretero‐Rey, María F. Garcia‐Garrido, Juan F. López Téllez, Inmaculada Jiménez‐Recuerda, Cristina A. Muñoz de Leon López, Zafar U. Khan

**Affiliations:** ^1^ Laboratory of Neurobiology CIMES University of Malaga Malaga Spain; ^2^ Department of Medicine Faculty of Medicine University of Malaga Malaga Spain; ^3^ CIBERNED Institute of Health Carlos III Madrid Spain; ^4^ Present address: Brain Metastasis Group National Cancer Research Centre (CNIO) Madrid Spain; ^5^ Present address: Departmento de Biología celular, Genética y Fisiología Universidad de Málaga Málaga Málaga Spain

**Keywords:** brain memory circuit activation, lesions in perirhinal cortex, memory deficits, recovery of memory dysfunctions, regulator of G protein signaling

## Abstract

**Aims:**

Ventral pathway circuits are constituted by the interconnected brain areas that are distributed throughout the brain. These brain circuits are primarily involved in processing of object related information in brain. However, their role in object recognition memory (ORM) enhancement remains unknown. Here, we have studied on the implication of these circuits in ORM enhancement and in reversal of ORM deficit in aging.

**Methods:**

The brain areas interconnected to ventral pathway circuits in rat brain were activated by an expression of a protein called regulator of G‐protein signaling 14 of 414 amino acids (RGS14_414_). RGS14_414_ is an ORM enhancer and therefore used here as a gain‐in‐function tool. ORM test and immunohistochemistry, lesions, neuronal arborization, and knockdown studies were performed to uncover the novel function of ventral pathway circuits.

**Results:**

An activation of each of the brain areas interconnected to ventral pathway circuits individually induced enhancement in ORM; however, same treatment in brain areas not interconnected to ventral pathway circuits produced no effect. Further study in perirhinal cortex (PRh), area V2 of visual cortex and frontal cortex (FrC), which are brain areas that have been shown to be involved in ORM and are interconnected to ventral pathway circuits, revealed that ORM enhancement seen after the activation of any one of the three brain areas was unaffected by the lesions in other two brain areas either individually in each area or even concurrently in both areas. This ORM enhancement in all three brain areas was associated to increase in structural plasticity of pyramidal neurons where more than 2‐fold higher dendritic spines were observed. Additionally, we found that an activation of either PRh, area V2, or FrC not only was adequate but also was sufficient for the reversal of ORM deficit in aging rats, and the blockade of RGS14_414_ activity led to loss in increase in dendritic spine density and failure in reversal of ORM deficit.

**Conclusions:**

These results suggest that brain areas interconnected to ventral pathway circuits facilitate ORM enhancement by an increase in synaptic connectivity between the local brain area circuits and the passing by ventral pathway circuits and an upregulation in activity of ventral pathway circuits. In addition, the finding of the reversal of ORM deficit through activation of an interconnected brain area might serve as a platform for developing not only therapy against memory deficits but also strategies for other brain diseases in which neuronal circuits are compromised.

## Graphical abstract

An increase in synaptic connectivity between each of the brain areas (perirhinal cortex is shown graph as an example) that are interconnected with ventral pathway circuits and ventral pathway circuits produces object recognition memory (ORM) enhancement and causes a reversal ORM deficit.

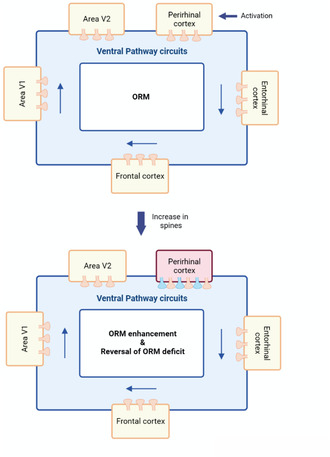

## 1 INTRODUCTION

The ventral pathway, which is also called as “what” pathway, begins in area V1 of visual cortex, passes through area V2, area V4, and temporal cortical areas, and finally converges into medial temporal and frontal lobes.^1^ It is thought that these interconnected brain areas that constitute ventral pathway circuits are crucial for object information processing and object recognition. However, within the brain areas that participate in the ventral pathway, perirhinal cortex (PRh) has been shown to play a vital role in the object recognition memory (ORM).^2^, ^3^ Lesions in this brain area deteriorate the ability of rats^4^, ^5^, ^6^ and monkeys^7^, ^8^, ^9^, ^10^ to perform on ORM tasks. The crucial role of PRh in ORM was further confirmed by receptor antagonist infusions in rats^11^, ^12^ and monkeys^13^ and by electrophysiological recordings in neuronal populations that appear to encode recognition memory in rats^14^, ^15^ and primates.^16^, ^17^ Therefore, the integrity of PRh is crucial for normal ORM function. However, we recently found that an enhancement in ORM, a paradigm that can serve as a strategy to combat memory deficits, is not dependent on PRh^18^ and ORM enhancement can be achieved by the activation of area V2 of visual cortex, a brain area that similar to PRh, is also interconnected to ventral pathway circuits.^18^, ^19^ An overexpression of a protein named RGS14_414_, which is an ORM enhancer,^19^ in the area V2 restored ORM in ORM‐deficient PRh‐lesioned rats and nonhuman primates.^18^ This finding led us to hypothesize that the overexpression RGS14_414_ into area V2 might enhance synaptic connectivity between area V2 local circuits and ventral pathway circuits and ultimately cause an activation in ventral pathway circuits. Thus, ORM enhancement may rely more on ventral pathway circuits than an individual brain area, such as PRh. If this concept is accurate, an enhancement in synaptic connectivity by overexpression of RGS14_414_ in any one of the interconnected brain areas might not only produce ORM enhancement similar to that observed in area V2, but also induce a reversal of the ORM deficit observed during normal aging. We have tested this idea here by examining whether RGS14‐mediated activation of various brain areas interconnected with ventral pathway circuits and distributed across occipital, medial temporal, and frontal lobes can induce ORM enhancement, and furthermore explored on the individual role of PRh, area V2, and frontal cortex (FrC) brain areas in facilitation of ORM enhancement and on the relevancy of these areas in RGS14‐mediated reversal of ORM deficit in aging.

## 2 MATERIALS AND METHODS

### 2.1 Animals

Wistar Han rats (research resource identifier (RRID), RGD_2308816) obtained from Charles River were used for this study. All procedures were performed in accordance with the guidelines of the Institutional Animal Care and Use Committee (IACUC) of the University of Malaga. Protocols (CEUMA 32‐2016‐A and 7‐2017‐A) for performing the experiments were approved by the IACUC of University of Malaga. One to two rats were housed per cage, and they were housed in a temperature‐regulated (20 ± 2°C) room under a 12‐h light/dark cycle. Drinking water and food were available ad libitum. The animals were acclimatized to the room for at least 1 week before starting the experiments, which took place during the light phase. All possible measures are taken to maintain the good health and well‐being of animals. Both departmental and veterinary staffs closely monitor animals and test routinely for the infection and diseases. Anesthetics were used in accordance with the guidelines of the IACUC of the University of Malaga to minimize suffering of animals during the surgery as well as the sacrifice.

In this study, rats of 3 months (young) and 18 months (aged) of age were used for the experiments and 1–2 rats were housed in each cage. A total of 584 rats were used where 62 rats were for untreated group (54 young and 8 aged), 117 rats were for vehicle treated group (93 young and 24 aged), 120 rats were for vehicle + Ox7 group (all young), 117 rats were for RGS14_414_ treated group (93 young and 24 aged), 120 rats for RGS14_414_ + Ox7 group (all young), and 48 rats for RGS14 + shRNA group (all aged).

### 2.2 Lentivirus preparation and delivery

**FIGURE 1:**
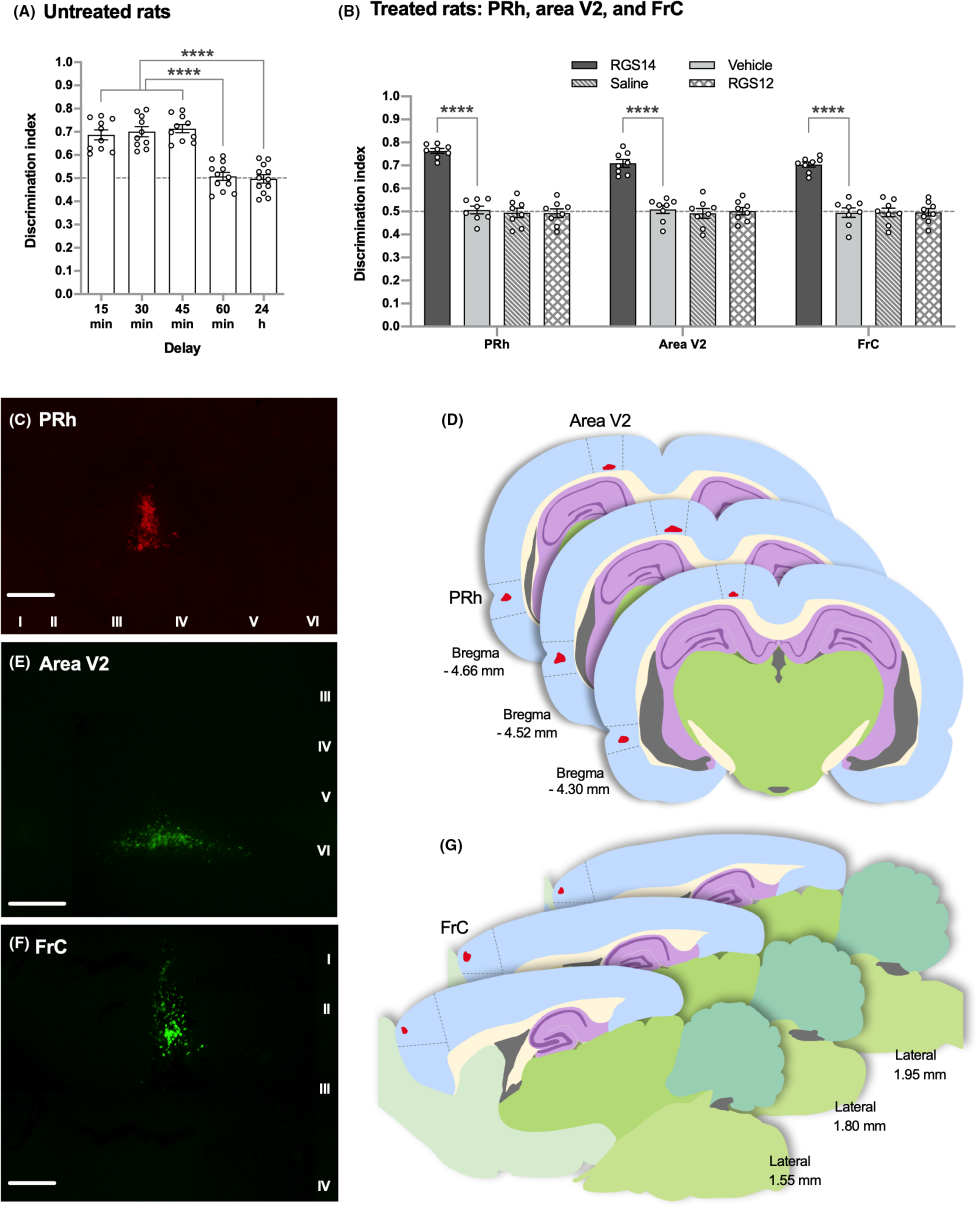
Activation of either PRh, area V2, or FrC by RGS14_414_ gene treatment induces ORM enhancement. (A) Three‐month‐old normal untreated rats were able to retain object information in memory for 15, 30, and 45 min; however, they were unable to retain such information after 60 min and 24 h. n = 10–12. (B) RGS14_414_ gene treatment either in PRh, area V2 or in FrC induced ORM enhancement that could be observed after 24 h, and treatment with vehicle, saline, or RGS12, another gene from the same family, caused no effect. *n* = 8. (C, E and F) Three images on the left show the immunolabeled RGS14_414_ protein in red in PRh area in C or green in area V2 and FrC in (E, F), respectively. Cortical layers are indicated in below of the image in C or right of the image in (E, F). Scale bars in all three images are 200 μm. (D, G) Drawings showing the localization of the RGS14_414_ protein (red) obtained after the analysis of serial coronal sections (in D) and sagittal sections (in G) from 5 RGS14_414_‐treated animals and representing the maximum expansion area all three sections of PRh and area V2 (in D) and FrC (in G). Dotted lines across panels (A, B) indicate the threshold at which (0.5 DI and below) the animals were unable to retain object information in memory. ****(one‐way ANOVA with post hoc Tukey's test in (A) and two‐way ANOVA with post hoc Sidak's test in (B), *p* < 0.0001).

Lentivirus preparation and the delivery into brain was done as earlier.^20^, ^21^ The cDNA of human RGS14 (GenBank accession number AY987041) was cloned into the pLenti6/Ubc/V5‐DEST Gateway vector (Thermo Fisher Scientific; catalog number V49910), and RGS14 lentivirus was produced according to the protocols of the ViraPower Lentiviral Expression System (Thermo Fisher Scientific). Vehicle lentivirus (vehicle) was prepared using vector alone. For the lentivirus delivery, rats were anesthetized with the sevoflurane system (induction at 5% sevoflurane + 1 L/min O_2_ and maintenance at 2% sevoflurane + 0.4 L/min O_2_) and placed in a stereotaxic frame according to the coordinates obtained from Stereotaxic Coordinates of the Rat Brain by Paxinos and Watson (fourth edition).^22^ The coordinates of the injection site in distinct brain areas are described in the Table 1. A total volume of 2 μL of RGS14_414_ lentivirus from a stock titer of 2.3 × 10^7^ TU/mL was injected bilaterally (1 μL in each hemisphere) through a 30‐gauge stainless steel internal cannula. During surgery, animal body temperature was maintained with an electric blanket. After surgery, animals were treated daily for 5 days with local antibiotic (as Dermocan manufactured by Fatro) application on the incision and 150 μL intraperitoneal injection of Meloxicam analgesic (as Metacam 5 mg/mL manufactured by Boehringer Ingelheim), and then they were left until the experiments started. Behavioral tests were performed 21 days after injection. To evaluate the surface area affected by RGS14_414_ lentivirus injection in the PRh, the brains of rats from Figure 1B were processed after behavioral studies and the results are shown in Figure 1.

**TABLE 1:** Coordinates of the brain areas for the injection of RGS14_414_.

Injection site	Coordinates (mm from the bregma)
Perirhinal cortex	AP −4.52, ML ±6.7, DV −4.75
Area V2	AP −4.30, ML ±2.1, DV −1.7
Frontal cortex	AP +4.70, ML ±2.2, DV −1.0
Area V1	AP −6.30, ML ±3.0, DV −1.3
Parietal cortex	AP −4.30, ML ±3.2, DV −1.1
Insular cortex	AP +1.2, ML ±5.0, DV −4.0
Thalamus	AP −3.60, ML ±2.1, DV −5.1
Basolateral amygdala	AP −3.14, ML ±4.9, DV −7.6
Entorhinal cortex	AP −5.2, ML ±7.0, DV −5.4

### 2.3 ORM test

The ORM test was performed as described previously.^19^, ^21^ Prior to the test, rats were handled for 8 min daily for five consecutive days and habituated to an open field apparatus (100 × 100 × 50 cm) for 12 min on the following 2 days. On the day of the experiment, for the object exposure session, the rats were placed in the same open field apparatus with 2 objects of identical shapes and were allowed to freely explore the objects for 3 min. After a delay from 15 min to 24 h, the ORM status of each animal was tested with one previously presented (familiar shape) object and a novel object with a different shape. The objects used in the study were plain bottles or containers of different shapes made of plastic or glass. The objects were similar in size to the rats such that the rats were unable to sit on or topple them. The objects were selected prior to ORM test according to two criteria: (a) the rats did not show a preference between novel and familiar objects in preference test experiments and (b) after exposure to the objects for 3 min, the rats were unable to recall them after a delay of 24 h. The location of the novel object was changed randomly between the left and right sides, which are the same fixed locations for two objects used during object exposure session. The open‐field apparatus and the objects were cleaned after each session. Objects shown previously were never presented to the same animal in any future sessions. The sessions were video recorded. The exploration time was calculated from the video by two independent researchers without knowledge of the animal conditions and then was averaged. Exploration time was defined only as an animal was touching the object with its nose. Standing on or using the object as a support was not considered exploration. The average total exploration time of the objects (familiar + novel) during the ORM test session for the vehicle and RGS14_414_ lentivirus‐treated animals was 31.76 ± 2.86 s and 30.93 ± 3.01 s, respectively.

**FIGURE 2:**
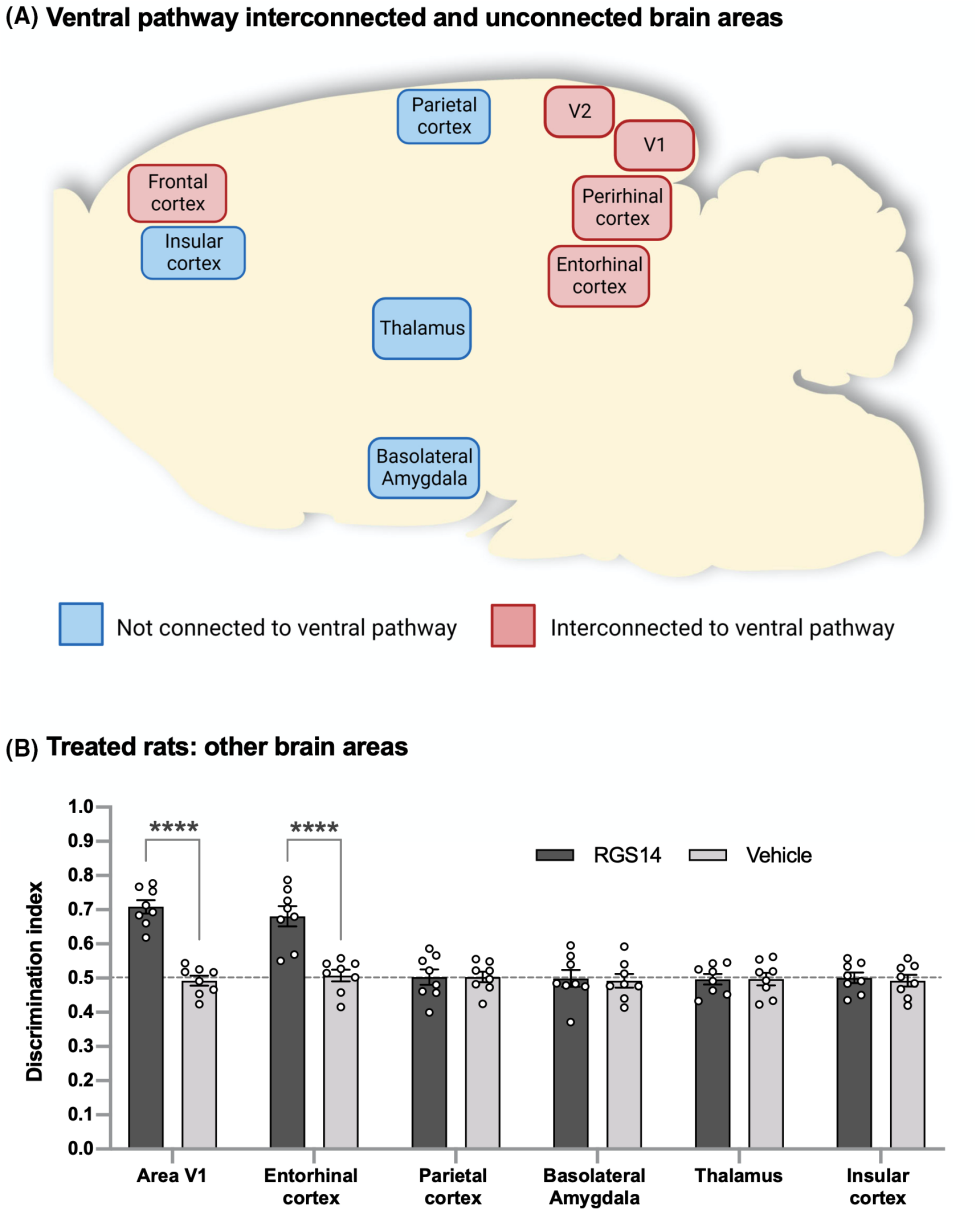
Activation of brain areas interconnected to ventral pathway circuits induces ORM enhancement. (A) Brain areas interconnected (pink) as well as not interconnected (blue) with ventral pathway circuits are shown according to their localization in the rat brain. V1 represents area V1 of visual cortex and V2 represents area V2 of visual cortex. (B) Similar to PRh, area V2, and FrC in Figure 1, RGS14_414_ gene treatment in area V1 or entorhinal cortex, which are also brain areas that are interconnected to ventral pathway circuits, induced an enhancement in ORM. However, same treatment in parietal cortex, basolateral amygdala, thalamus or insular cortex, which are brain areas that are not interconnected to ventral pathway circuits, produce no effect on ORM and the ORM levels were same as in rats treated with vehicle. *n* = 8. Dotted lines across panel (B) indicates the threshold at which (0.5 DI and below) the animals were unable to retain object information in memory. ****(two‐way ANOVA with post hoc Sidak's test, *p* < 0.0001).

**FIGURE 3:**
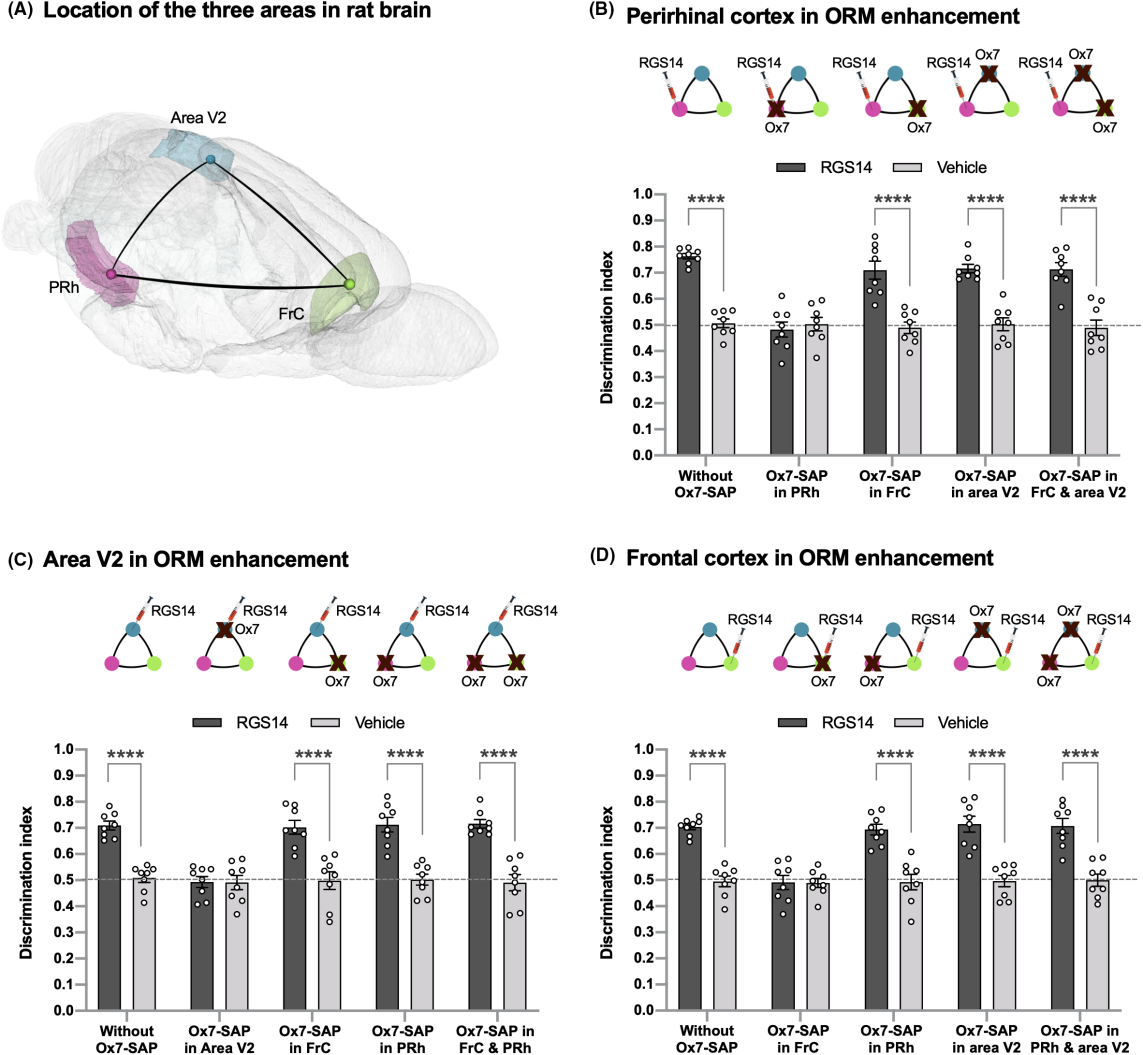
ORM enhancement seen after activation of either PRh, area V2 or FrC is independent to other two brain areas. (A) Localization of three brain areas, Prh, area V2 and FrC, included in the study are shown in rat brain. (B) RGS14_414_ gene treatment in PRh induced ORM enhancement; however, elimination of PRh neurons by immunotoxin Ox7‐SAP abrogated this ORM enhancement. In contrast, when these RGS14‐treated rats in PRh were subjected to Ox7‐SAP treatment either individually in FrC and area V2 or concomitantly in both areas, they showed no change in ORM enhancement. *n* = 8. (C) RGS14_414_ treatment in area V2 caused ORM enhancement, whereas an elimination of area V2 neurons by Ox7‐SAP abolished this ORM enhancement. However, similar to as in PRh, Ox7‐SAP treatment in RGS14‐treated rats in area V2 either individually in FrC and PRh or simultaneously in both areas produced no alteration in ORM enhancement. *n* = 8. (D) RGS14_414_ gene treatment in FrC induced ORM enhancement and this ORM enhancement was abolished after elimination of FrC neurons by Ox7‐SAP treatment. However, same RGS14‐treated rats in FrC when subjected to Ox7‐SAP treatment either individually in PRh and area V2 or concomitantly showed no alteration in ORM enhancement. *n* = 8. Dotted lines across panels (B–D) indicate the threshold at which (0.5 DI and below) the animals were unable to retain object information in memory. ****(two‐way ANOVA with post hoc Sidak's test, *p* < 0.0001).

Discrimination index (DI) data are presented in Figures 1, 2, 3, 5, and 6. The DI was calculated by dividing the time spent exploring the novel object by the total exploration time (familiar object + novel object). A DI equal to or less than 0.5 indicated that the animals were unable to retain object information in memory because they explored both the familiar and novel objects for equal amounts of time (50% for the familiar object and 50% for the novel object), whereas a DI above 0.66 indicated that the animals were able to successfully retain information about the object in memory because they spent more than 66% of the total time exploring novel objects and less than 34% of the time exploring familiar objects.

### 2.4 Immunohistochemistry

Immunohistochemistry was performed as described previously.^19^, ^23^ In brief, after termination of the behavioral tests, the rats were perfused transcardially with a fixative containing 4% paraformaldehyde, 1.37% L‐lysine, and 0.21% meta‐periodate and cryoprotected with 30% sucrose. Sagittal brain sections with a thickness of 30 μm were incubated overnight at 4°C with an affinity‐purified rabbit anti‐RGS14 antibody (1:30 dilution) that was prepared in our laboratory.^19^ The sections were then incubated for 90 min with either Alexa Fluor 488 or Alexa Fluor 647 goat anti‐rabbit IgG (1:1000 dilution; A32731 (for 488) and A21246 (for 647); Thermo Fisher Scientific), and immunofluorescence labeling was detected by confocal microscopy.

### 2.5 Ox7‐SAP injection

The injection of Ox7‐SAP (0.2 μg in 1 μL; IT‐02; Advanced Targeting Systems) into PRh, area V2, and FrC was performed in a manner similar to that described in the Lentivirus preparation and delivery section, using the coordinates as in Table 1. The extent of damage in the brains of these animals was analyzed after staining the brain sections with cresyl violet. Analysis of the brains of rats treated with Ox7‐SAP in PRh, area V2, and FrC showed substantial neuronal damage in all three brain areas (Figure S1).

### 2.6 Neuronal arborization

Neuronal arborization study was performed as earlier.^20^ Briefly, brains of RGS14‐rats treated either in PRh, area V2, or in FrC were processed for Golgi‐Cox staining using a Rapid GolgiStain kit (FD Neurotechnologies; catalog number PK401) following the protocol from the manufacturer. All procedures were performed protected from light. A 180‐μm section was prepared with a cryostat, and after staining, the sections were mounted with Permount mounting medium (Thermo Fisher Scientific; catalog number SP15‐100). Pyramidal neurons were analyzed in the area of injection under a DM IRE2 microscope (Leica Microsystems) using Leica MM AF software, version 1.6.0 (Leica Microsystems). A total of 38–44 pyramidal neurons from seven animals were studied in the vehicle lentivirus‐treated group, and 45–49 pyramidal neurons from seven animals were studied in the RGS14_414_ lentivirus‐treated group. These pyramidal neurons were randomly selected. Neuronal tracing, Sholl analysis for neuronal arborization, total neuronal cable length, and neuronal branching studies were done using ImageJ program. Spines were counted from the apical dendrites of pyramidal neurons according to their physical appearance into the thin, mushroom and stubby groups, as described previously.^24^ However, there was no difference between vehicle and RGS14‐treated animals in any of the spine groups and therefore, all spine groups within each treatment were combined.

### 2.7 Knockdown of 14‐3‐3ζ gene in rats

Small hairpin RNA (shRNA) adeno‐associated virus (AAV) particles of the rats 14‐3‐3ζ gene (GenBank accession NM_013011.3) were purchased from GeneCopoeia (catalog number AA10‐RSE049324‐AVE001‐200). Control shRNA AAV particles were also from GeneCopoeia (catalog number AC202). Rats were injected stereotaxically with a total of 2 μL containing 1 × 10^10^ GC AAV particles of shRNA of the 14‐3‐3ζ gene or control shRNA bilaterally (1 μL in each hemisphere) in either the PRh, area V2 or the FrC at coordinates similar to those described above in Table 1 of Lentivirus preparation and delivery section. Seven days after the injection, both groups of shRNA rats were injected with the lentivirus of RGS14_414_ gene. These rats were then used for experiments after 28 days of the shRNA injection, which is 21 days after RGS14_414_ gene treatment. In initial experiments to determine the efficacy of 14‐3‐3ζ gene knockdown with western blot method, we observed that a treatment with the shRNA of 14‐3‐3ζ in normal rats reduced 14‐3‐3ζ protein expression to 24.37 ± 2.49% (*n* = 4; two experiments from each of the two sets of brain homogenate prepared from a pool of four animal brain in each set) and in RGS14‐treated rats reduced 14‐3‐3ζ protein expression to 26.94 ± 2.43% (*n* = 6; three experiments from each of the two sets) (Figure S2).

### 2.8 Statistics

**FIGURE 4:**
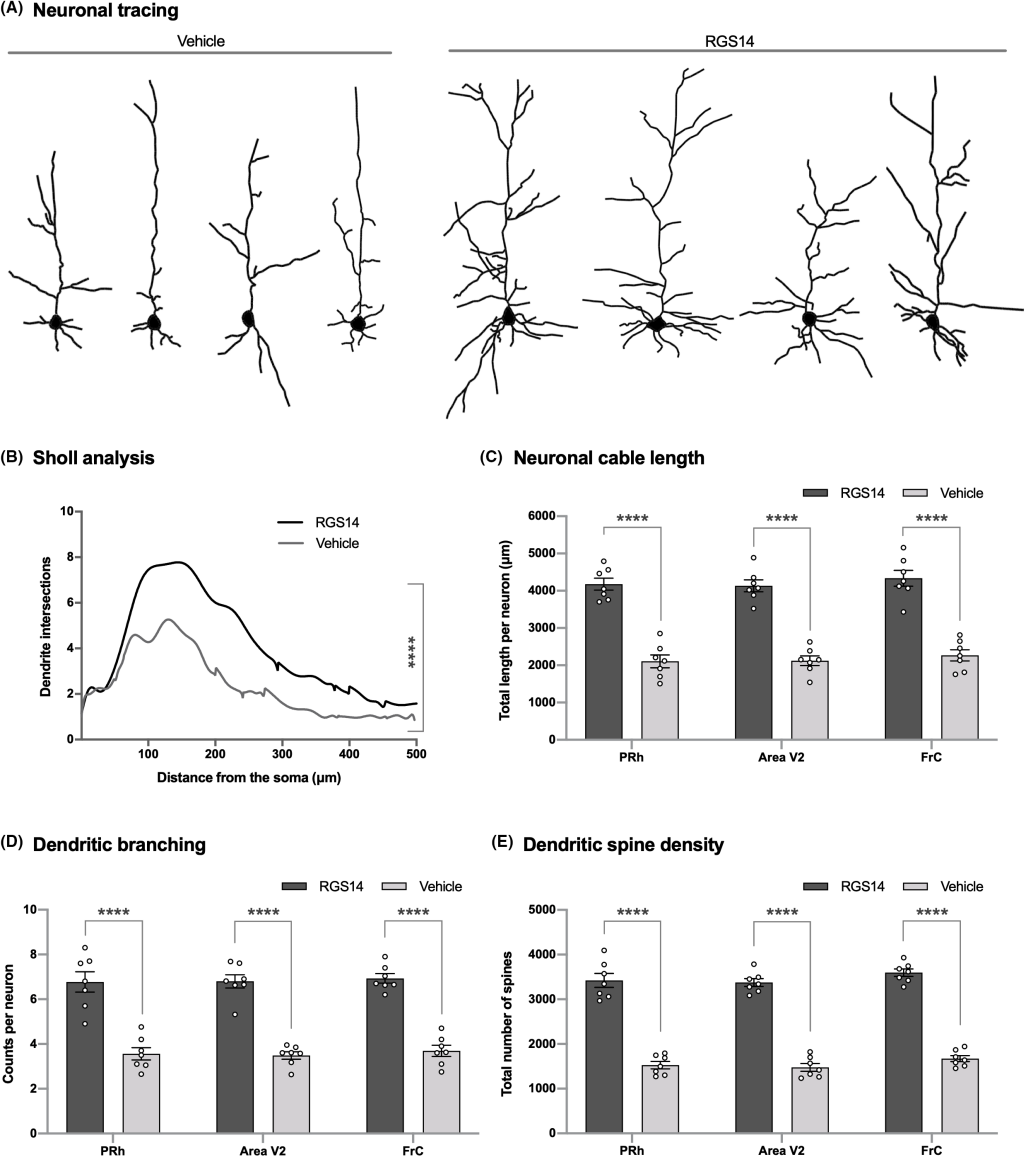
RGS14_414_ gene treatment in PRh, area V2 or FrC induces neuronal arborization in pyramidal neurons. (A) Neuronal tracings of few examples of pyramidal neurons are shown and an increase in neuronal arbors was observed in rats treated with RGS14_414_. (B) Sholl analysis of pyramidal neurons showed an increase in neuronal arborization in RGS14‐treated rats. *n* = 7. (C) An increase in total neuronal cable length in pyramidal neurons was observed in RGS14‐rats treated either in PRh, area V2 or in FrC was observed. *n* = 7. (D) RGS14‐rats treated either in PRh, area V2 or in FrC showed increase in the number of dendritic branches in pyramidal neurons. *n* = 7. (E) Coinciding with the increase in dendritic branching, an upsurge in total spine number was observed in RGS14‐rats treated in PRh, area V2 or FrC. *n* = 7. ****(two‐tailed unpaired *t* test in (B); two‐way ANOVA with Sidak's post hoc test in (C–E), *p* < 0.0001).

**FIGURE 5:**
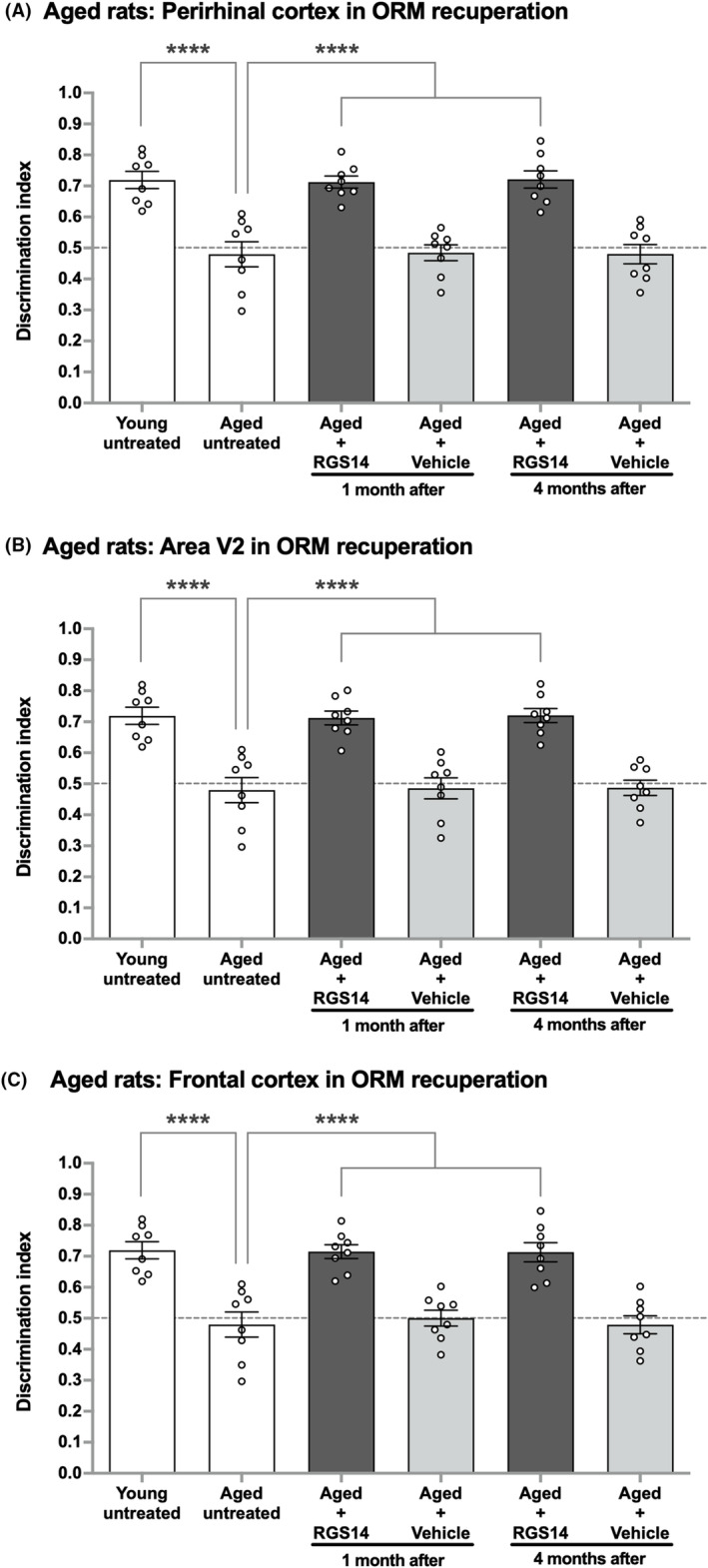
Reversal of ORM deficit in aging rats after RGS14_414_ treatment either in PRh, area V2 or in FrC. (A) When young untreated rats were exposed to an object, they could retain the object information in memory; however, aged untreated rats were unable to retain this information. RGS14_414_ gene treatment in area PRh of these ORM‐deficient aged rats led to full recovery in ORM tested 1 month after the treatment (Aged + RGS14). In contrast, vehicle treatment did not produce any effect (Aged + Vehicle). This recuperated ORM in RGS14‐treated animals was maintained even 4 months after the treatment. *n* = 8. (B) Similar to PRh, RGS14_414_ gene treatment in area V2 of the ORM‐deficient aged rats caused complete reversal in ORM deficit evaluated after 1 month of the treatment (Aged + RGS14), and this recuperated ORM persisted 4 months after the treatment. *n* = 8. (C) Similarly, when ORM‐deficient aged rats were treated with RGS14_414_ gene in FrC, a full recovery in ORM was observed after 1 month of the treatment and the recuperated ORM was maintained 4 months after the treatment (Aged + RGS14). *n* = 8. Dotted lines across panels indicate the threshold at which (0.5 DI and below) the animals were unable to retain object information in memory. ****(one‐way ANOVA with Tukey's post hoc test, *p* < 0.0001).

**FIGURE 6:**
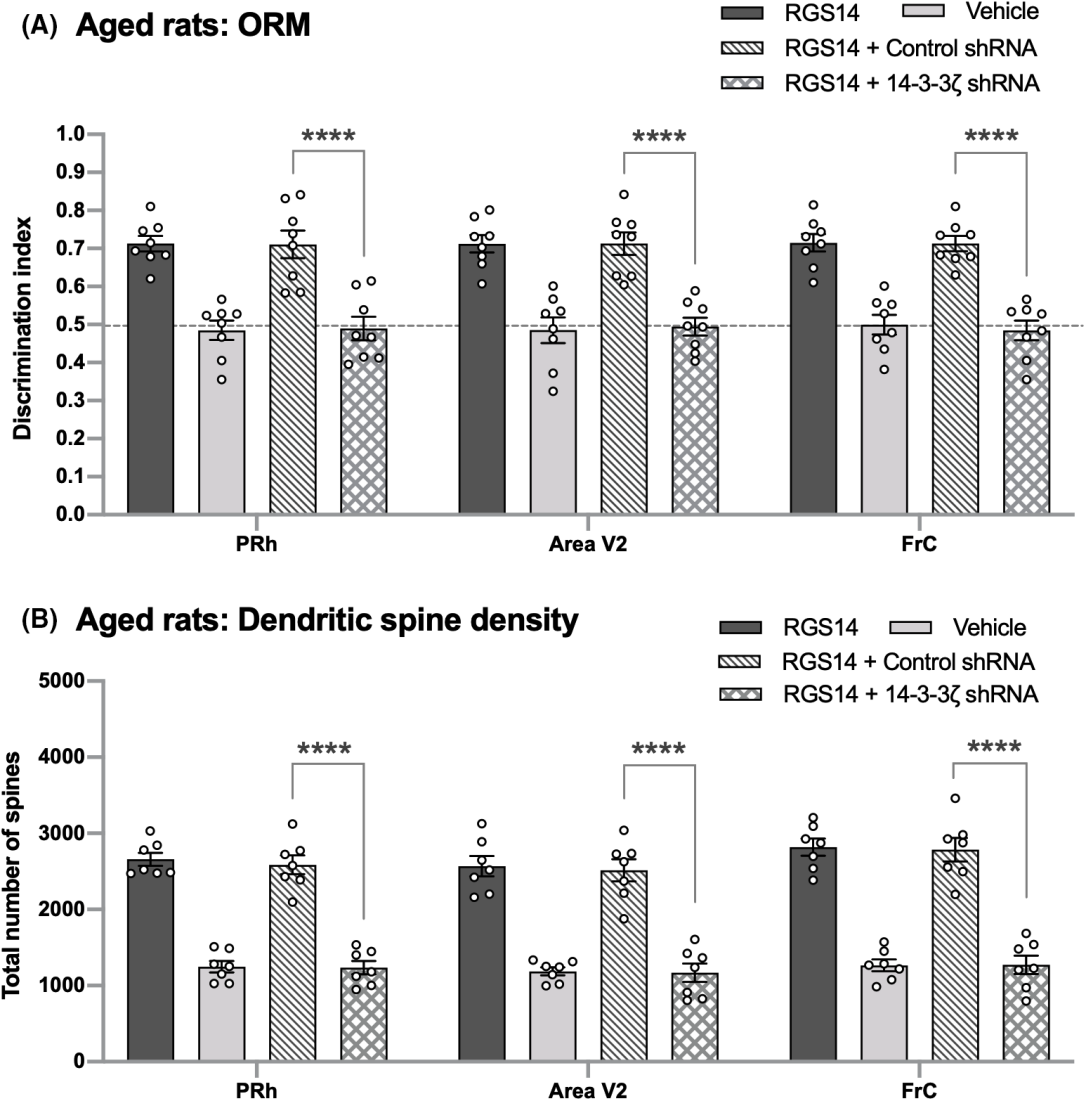
Blockade of RGS14_414_ activity leads to failure in reversal of ORM deficit and loss in increase in spine density in aging rats. (A) RGS14_414_ gene treatment either in PRh, area V2, or in FrC caused reversal in ORM deficit in aging rats; however, vehicle treatment had no effect in all three brain areas. Further, a treatment of these rats with shRNA of 14‐3‐3ζ, which is expected to suppress the effect of RGS14_414_, led to failure in reversal of ORM deficit (RGS14 + 14‐3‐3ζ shRNA) in all three brain areas. *n* = 8. (B) The failure in reversal of ORM deficit in RGS14 + 14‐3‐3ζ shRNA rats was accompanied by the loss in increase in dendritic spine number of pyramidal neurons. This effect was not observed when RGS14‐rats treated either in PRh, area V2 or in FrC were subjected to treatment with control shRNA (RGS14 + Control shRNA). *n* = 8. Dotted lines across panel A indicate the threshold at which (0.5 DI and below) the animals were unable to retain object information in memory. ****(two‐way ANOVA with Sidak's post hoc test, *p* < 0.0001).

The results were plotted, and statistical significance was evaluated using Prism 8. The data used to construct Figures 1, 2, 3, 4, 5, 6 passed the normality test done by the Shapiro–Wilk normality test, and they showed *p* values ranged from 0.063 to 0.989. The equality of group variances was tested by the Brown‐Forsythe test, and the *p* values ranged from 0.062 to 0.952. More than two group comparisons with single variables were analyzed using one‐way ANOVA with Tukey's post hoc test. For multiple group comparisons with more than one variable, two‐way ANOVA with the Sidak's post hoc test was used. All data in the figures are presented as the mean ± SEM values.

## 3 RESULTS

### 3.1 Brain areas interconnected to ventral pathway are capable of ORM enhancement

Similar to earlier, a lentivirus containing the RGS14_414_ gene was delivered into a brain areas of Wistar Han rats to induce expression of the RGS14_414_ protein, and the memory status of these animals was evaluated by ORM test 21 days after the treatment.^20^, ^25^ We observed that when normal untreated rats of 3 months old were exposed to an object for 3 min, they were able to retain information about the object in memory 15, 30 or 45 min after the object exposure but not after 60 min and 24 h (Figure 1A; one‐way ANOVA, *F*(4, 49) = 33.51; 15, 30 and 45 min versus 60 min or 24 h, Tukey's multiple comparisons test, *p* < 0.0001). However, rats treated with the RGS14_414_ gene either in PRh, area V2, or in FrC, which are brain areas that are interconnected to ventral pathway circuits, were able to keep the same object information in memory 24 h after the object exposure (Figure 1B; two‐way ANOVA, *F*(3, 84) = 129.70; RGS14 versus vehicle, Sidak's multiple comparisons test, *p* < 0.0001), and this effect of RGS14_414_‐gene treatment on memory enhancement was long‐lasting (Figure S3). Treatment of rats with lentivirus of empty plasmid (vehicle), saline solution, or even lentivirus of RGS12, a protein that belongs to the same family as RGS14_414_, did not produce any effect on ORM (Figure 1B; two‐way ANOVA, *F*(3, 84) = 129.70; vehicle versus saline or RGS12, Sidak's multiple comparisons test, *p* > 0.99), and the performance of these rats in the test was similar to that of untreated rats (24 h in Figure 1A). These results suggest that when PRh, area V2, and FrC are individually activated by RGS14_414_ gene treatment, they can induce ORM enhancement. Furthermore, after termination of the behavioral studies, the brains of the rats were processed to evaluate the affected surface area after RGS14_414_ lentivirus treatment. Images of brain sections after immunostaining with antibodies specific for RGS14 are shown in Figure 1C for PRh, Figure 1E for area V2, and Figure 1F for FrC; and a depiction of brain sections after analysis of serial sections, showing maximum expansion of the RGS14_414_ protein in brain areas are in Figure 1D for PRh and area V2 (drawings in red color) and Figure 1G for FrC (drawings in red color). The results of immunostaining studies in all three brain areas indicate that RGS14_414_ protein expression in each corresponding brain areas was confined to the target area.

The capacity of PRh, area V2, and FrC in ORM enhancement imply that these brain areas interact directly with the ventral pathway circuits that are liable for object recognition to induce the effect on ORM. According to this concept, brain areas that are not directly interconnected to ventral pathway circuits should not cause the RGS14_414_‐mediated ORM enhancement. Therefore, to further explore this hypothesis, we examined brain areas that are not interconnected to ventral pathway circuits and additionally, brain areas other than above three that are interconnected to this pathway (Figure 2A). Indeed, we found that RGS14_414_ treatment in parietal cortex, a brain area known to be interconnected to dorsal pathway and not to the ventral pathway and is involved in spatial memory,^1^ produced no effect on ORM (Figure 2B; two‐way ANOVA, *F*(5, 42) = 11.15; vehicle versus RGS14, Sidak's multiple comparisons test, *p* > 0.99). In addition to parietal cortex, a treatment with RGS14_414_ gene in basolateral amygdala, thalamus, and insular cortex, which are other brain areas that are not interconnected to ventral pathway circuits, caused no effect on ORM either. In contrast, treatment with the RGS14_414_ gene in area V1, or entorhinal cortex, both brain areas are interconnected to the ventral pathway, induced ORM enhancement (Figure 2B; two‐way ANOVA, *F*(5, 42) = 11.15; vehicle versus RGS14, Sidak's multiple comparisons test, *p* < 0.0001). Thus, altogether, our results of functional study using the paradigm of RGS14_414_‐mediated ORM enhancement indicate that brain areas that are interconnected to ventral pathway circuits, are apt for ORM enhancement, and the brain areas that are not directly connected to ventral pathway circuits, are not.

### 3.2 ORM enhancement induced by a brain area is not dependent on other brain areas

Next, we wanted to examine whether a brain area interconnected to ventral pathway circuits can induce RGS14_414_‐mediated ORM enhancement without the implication of other brain areas or not. For this study, we have focused on PRh, area V2, and FrC (Figure 3A), brain areas that have been shown to be involved in ORM and are able to induce RGS14_414_‐mediated ORM enhancement. The plan of this study was to treat one of these three brain areas with RGS14_414_ gene and then analyze the effect of neuronal loss in the other two brain areas on RGS14_414_‐mediated ORM enhancement. To eliminate the neurons, we have used Ox7‐SAP, which is a saporin‐based immunotoxin that causes selective eradication of neurons and does not affect other brain structures or passing nerve fibers.^19^, ^26^, ^27^ We found that when rats treated with RGS14_414_ in PRh area (RGS‐PRh rats) were subjected to an injection of Ox7‐SAP in the same brain area (PRh), the ORM enhancement in RGS‐PRh rats was completely abolished (Figure 3B; Ox7‐SAP in PRh; two‐way ANOVA, *F*(4, 35) = 13.37; vehicle versus RGS14, Sidak's multiple comparisons test, *p* = 0.99), and the performance of these RGS‐PRh rats reached to a level of those of vehicle‐treated animals. However, the injection of Ox7‐SAP either in FrC, area V2, or both areas simultaneously in RGS‐PRh rats did not cause any effect on RGS14_414_‐mediated ORM enhancement (Figure 3B; Ox7‐SAP in FRC, area V2 or FrC & area V2; two‐way ANOVA, *F*(4, 35) = 13.37; vehicle versus RGS14, Sidak's multiple comparisons test, *p* < 0.0001). Similar to PRh, a treatment with RGS14_414_ in area V2 (RGS‐V2 rats) induced ORM enhancement (Figure 3C; without Ox7‐SAP; two‐way ANOVA, *F*(4, 35) = 13.37; vehicle versus RGS14, Sidak's multiple comparisons test, *p* < 0.0001) and an injection of Ox7‐SAP in the same area (area V2) of these RGS‐V2 rats caused complete loss in ORM enhancement (Figure 3C; Ox7‐SAP in Area V2; two‐way ANOVA, *F*(4, 35) = 13.38; vehicle versus RGS14, Sidak's multiple comparisons test, *p* > 0.99). However, in contrary, an injection of Ox7‐SAP in FrC, PRh, or both areas simultaneously in RGS‐V2 rats produced no effect on RGS14_414_‐mediated ORM enhancement (Figure 3B; Ox7‐SAP in FRC, PRh or FrC & PRh; two‐way ANOVA, *F*(4, 35) = 13.38; vehicle versus RGS14, Sidak's multiple comparisons test, *p* < 0.0001). In addition to PRh and area V2, we found similar results in FrC area. When rats treated with RGS14_414_ in FrC area (RGS‐FrC rats) were injected with Ox7‐SAP in the same brain area (FrC), a complete loss in RGS14_414_‐mediated ORM enhancement was observed in RGS‐FrC rats (Figure 3D; Ox7‐SAP in FrC; two‐way ANOVA, *F*(4, 35) = 7.53; vehicle versus RGS14, Sidak's multiple comparisons test, *p* > 0.99), whereas the injection of Ox7‐SAP in PRh, area V2, or both areas simultaneously in RGS‐FrC rats did not produce any effect on RGS14_414_‐mediated ORM enhancement (Figure 3D; Ox7‐SAP in PRh, area V2 or PRh & area V2; two‐way ANOVA, *F*(4, 35) = 7.53; vehicle versus RGS14, Sidak's multiple comparisons test, *p* < 0.0001). The results from all three brain areas suggest that each brain area is independently able to induce ORM enhancement, and that the ORM enhancement rely more on ventral pathway circuits than an individual brain area. Further analysis after the termination of behavioral studies of the brains of rats treated with Ox7‐SAP in PRh, area V2, and FrC showed substantial neuronal damage in all three brain areas (Figure S1).

### 3.3 RGS14_414_ treatment promotes neuronal structural plasticity and increase in spines

The capacity of brain areas interconnected to ventral pathway circuits in RGS14‐mediated ORM enhancement suggests a direct interaction of these brain areas with the passing by ventral pathway circuits, and it is likely that a treatment with RGS14_414_ gene might cause an increase in synaptic connectivity between the local circuits of interconnected brain area and the ventral pathway circuits. This theory of structural plasticity might also explain why a treatment with RGS14_414_ gene led to long‐lasting effect. Therefore, we next examined the structural change in pyramidal neurons, which are brain structures that are closely associated to synaptic plasticity and learning and memory.^28^ Rat brains obtained after 21 days of the RGS14_414_ treatment were processed for Golgi‐Cox silver staining and structural changes in pyramidal neurons were analyzed. We found a robust neuronal arborization in pyramidal neurons (Figure 4A), and further Sholl analysis revealed considerably higher neuronal arbors in those neurons (Figure 4B; two‐tailed unpaired t test; vehicle versus RGS14, *p* < 0.0001). Accordingly, an increase in total neuronal cable length was found in pyramidal neurons of rats treated with RGS14_414_ gene in PRh, area V2 or FrC (Figure 4C; two‐way ANOVA, *F*(1, 18) = 272.6; vehicle versus RGS14, Sidak's post hoc test, *p* < 0.0001). In addition, an analysis of branching showed an increase in dendritic branching of pyramidal neurons of rats treated with RGS14_414_ gene in PRh, area V2, or FrC (Figure 4D; two‐way ANOVA, *F*(1, 18) = 225.9; vehicle versus RGS14, Sidak's post hoc test, *p* < 0.0001). Nevertheless, the effect of RGS14_414_ gene treatment was more prominent in dendritic spines of pyramidal neurons where more than 2‐fold increase in the total number of dendritic spines was observed in rats treated with RGS14_414_ gene in PRh, area V2 or FrC (Figure 4E; two‐way ANOVA, *F*(1, 18) = 446.9; vehicle versus RGS14, Sidak's post hoc test, *p* < 0.0001). Thus, our results support the idea that an increase in synaptic connectivity between brain area interconnected to ventral pathway and ventral pathway circuits promotes ORM enhancement.

### 3.4 RGS14_414_ treatment in any of the three brain areas rescues ORM deficit in aging rats

Considering that each one of the three brain areas (PRh, area V2, and FrC) is independently able to induce ORM enhancement despite the substantial damage in other two brain areas, we next examined whether RGS14_414_ gene treatment in any of these three brain areas could reverse ORM deficit in normal aging, which is one of the most studied condition of memory deficits.^29^, ^30^ For this study, aged Wistar rats were treated with RGS14_414_ gene in PRh, area V2 or FrC and memory levels of these rats were determined 1 month and 4 months after the treatment. We observed that untreated young rats of 3 months of age (young untreated) were able to keep the information about an object in memory. However, when these rats reached 18 months of age (aged untreated), they were unable to recall the same information and showed noticeable decline in their ORM levels (Figure 5A–C; one‐way ANOVA, *F*(5, 42) = 19.45; young untreated versus aged untreated, Tukey's multiple comparisons test, *p* < 0.0001). A treatment of these ORM‐deficient aged rats with the RGS14_414_ gene either in PRh (Figure 5A; one‐way ANOVA, *F*(5, 42) = 19.45; aged untreated versus aged + RGS14, 1 month after, Tukey's multiple comparisons test, *p* < 0.0001), in area V2 (Figure 5B; one‐way ANOVA, *F*(5, 42) = 19.00; aged untreated versus aged + RGS14, 1 month after, Tukey's multiple comparisons test, *p* < 0.0001), or in FrC (Figure 5C; one‐way ANOVA, *F*(5, 42) = 17.78; aged untreated versus aged + RGS14, 1 month after, Tukey's multiple comparisons test, *p* < 0.0001) led to full recovery in ORM. The performance of aging rats treated with RGS14_414_ gene in all three brain areas reached to similar level as to those of young untreated animals, and the recovered ORM in aging rats was maintained even after 4 months of the treatment in PRh (Figure 5A; one‐way ANOVA, *F*(5, 42) = 19.45; aged untreated versus aged + RGS14, 4 months after, Tukey's multiple comparisons test, *p* < 0.0001), in area V2 (Figure 5B; one‐way ANOVA, *F*(5, 42) = 19.00; aged untreated versus aged + RGS14, 4 months after, Tukey's multiple comparisons test, *p* < 0.0001) or in FrC (Figure 5C; one‐way ANOVA, *F*(5, 42) = 17.78; aged untreated versus aged + RGS14, 4 months after, Tukey's multiple comparisons test, *p* < 0.0001). In contrast to the treatment with RGS14_414_, vehicle treatment in aging rats did not cause recovery in ORM, and the ORM levels of vehicle‐treated rats were similar to those of untreated aged rats. These results indicate that the increase in synaptic connectivity between the brain areas that are interconnected to ventral pathway and ventral pathway circuits could in fact induce reversal in ORM deficit in aging rats.

### 3.5 Suppression of RGS14_414_ activity causes failure in rescue of ORM deficit and loss in increase in spine density in aging rats

It has been shown that RGS14_414_ mediates its effect through the activation of 14‐3‐3ζ.^25^ Therefore, blocking the 14‐3‐3ζ activity in RGS14_414_‐treated aged rats should abolish both the reversal in ORM deficit and the increase in spine density. To evaluate the effect of 14‐3‐3ζ in RGS14‐mediated reversal of ORM deficit and increase in spine density, aged rats treated with RGS14_414_ either in PRh, area V2, or in FrC were subjected to treatment with shRNA of 14‐3‐3ζ with the goal of curbing the expression of 14‐3‐3ζ protein. In general, this shRNA treatment reduced the expression of 14‐3‐3ζ protein to 26.94 ± 2.43% in rats (Figure S2). First, we studied the effect of 14‐3‐3ζ shRNA treatment on reversal of ORM deficit. RGS14_414_ gene treatment either in PRh, area V2, or in FrC of ORM‐deficient aged rats led to full recovery in ORM, whereas vehicle treatment produced no effect at all (Figure 6A; PRh, area V2 or FrC; two‐way ANOVA, *F*(3, 63) = 65.22; vehicle versus RGS14, Sidak's multiple comparisons test, *p* < 0.0001), and this recovered ORM disappeared when they were treated with 14‐3‐3ζ shRNA (Figure 6A; PRh, area V2 or FrC; two‐way ANOVA, *F*(3, 63) = 65.22; RGS14 + Control shRNA versus RGS14 + 14‐3‐3ζ shRNA, Sidak's multiple comparisons test, *p* < 0.0001). In line with the reversal in ORM deficit after RGS14_414_ treatment in aging rats, we observed that a treatment with the RGS14_414_ gene either in PRh, area V2, or FrC brain areas induced a substantial increase in dendritic spine density (Figure 6B; PRh, area V2 or FrC; two‐way ANOVA, *F*(3, 54) = 210.6; vehicle versus RGS14, Sidak's multiple comparisons test, *p* < 0.0001) and the increase in dendritic spine density was abolished when these rats were treated with 14‐3‐3ζ shRNA (Figure 6B; PRh, area V2 or FrC; two‐way ANOVA, *F*(3, 54) = 210.6; RGS14 + Control shRNA versus RGS14 + 14‐3‐3ζ shRNA, Sidak's multiple comparisons test, p < 0.0001). In contrast, when same rats were subjected to treatment with control shRNA instead of 14‐3‐3ζ shRNA, they posed no effect on increase in synaptic density. The complete loss of recuperation in ORM deficit as well as increase in synaptic density after hampering RGS14_414_ effect by 14‐3‐3ζ knockdown suggests that the reversal in ORM deficit in aging rats is facilitated through the enhancement in synaptic connectivity between the brain areas interconnected to ventral pathway circuits and the ventral pathway circuits.

## 4 DISCUSSION

Our findings demonstrate that RGS14_414_ gene treatment in brain areas interconnected to ventral pathway circuits was independently able to induce ORM enhancement and that this treatment was adequate for the rescue of ORM deficit in aging rats. We further found that the reversal in ORM deficit was dependent on the neuronal structural plasticity in pyramidal neurons, which is a kind of change in brain that is long‐lasting and often produces long‐term effect. Additionally, pyramidal neurons are brain structures that innervate to other brain regions and primarily makes efferent synaptic connections for regulating brain functions.^28^ Therefore, an increase of more than two‐fold in dendritic spines of pyramidal neurons in PRh, area V2, and FrC is expected to cause synaptic reorganization in interconnecting network of these brain areas, and this increased synaptic connectivity can eventually provide a structural foundation for uplifting the activity of ventral pathway circuits. Considering that ventral pathway circuits are essential for object information processing and object recognition, and PRh, area V2, and FrC brain areas are the main contributors to these pathway circuits, a modulation in synaptic connectivity between any of these brain areas and passing by ventral pathway circuits could result in a considerable effect on ORM. It has been shown that memory is critically associated with synaptic remodeling and increase in synaptic connectivity,^31^, ^32^ and memory deficits, such as during the aging, is a consequence of reduced activity within the memory circuits.^33^, ^34^, ^35^ Consistent with this idea, poor performance in patients with memory deficiency has been shown to be associated with decreased functional activity in neural networks in the medial temporal lobe and neocortex.^33^, ^36^ Therefore, an increase in the activity of ventral pathway circuits might not only induce ORM enhancement but also cause reversal in ORM deficit. Indeed, our results affirm this assumption and show that RGS14_414_‐mediated increase in synaptic connectivity in either PRh, area V2 or FrC facilitates ORM enhancement and causes full recuperation in ORM deficit, and further blocking of the increase in spines led to failure in reversal of ORM deficit. Similar to ventral pathway circuits, enhancement in a different neuronal network connectivity in brain of patients with cognitive deficit by either transcranial direct current stimulation (tDCS) or repetitive transcranial magnetic stimulation (rTMS) also improved cognitive performance of those patients.^37^ Thus, an upregulation in activity of ventral pathway brain circuits might underlie not only the ORM enhancement but also the reversal in ORM deficit.

Normal aging rats without RGS14_414_ treatment showed noticeable ORM deficits, and in contrary to normal untreated young rats, they failed to retain the object information in memory. Nonetheless, considering the set up required for performing ORM study in our experimental conditions, it is not possible to correlate the ORM deficits in aging rats to any one of the brain areas studied here. However, in contrast to the ORM study, dendritic spine density in all three brain areas (PRh, area V2, and FrC) in aging rats without RGS14_414_ treatment was lower than in untreated young rats, and the reduction in dendritic spine density was at same the level in these brain areas. Thus, there were no selective loss in pyramidal spine density in any one of the brain areas that can be accounted for ORM deficits in aging rats. Instead, a same level of decrease in dendritic spine density in all three brain areas was observed. This result is not surprising because lesions studies together with studies in distinct brain areas interconnected to ventral pathway circuits in the current study suggest that ORM functions rely more on the ventral pathway circuits than on any individual brain area. In agreement with our results, it has been argued that memory deficits occur because of reduced activity within brain circuits.^33^, ^34^, ^35^ Hence, an activation in ventral pathway circuits, such as the treatment with RGS14_414_, is expected to improve ORM. Findings from our study indicate that both the ORM enhancement and recovery are most likely the product of activation of ventral pathway circuits, and that the activity of these circuits could be augmented by the activation of any one of the three brain areas (PRh, area V2, and FrC) interconnected to ventral pathway circuits. However, whether there are distinct mechanisms or subdomains within these brain areas that drive RGS14‐mediated ORM enhancement and recovery remains to be studied. Nevertheless, our results from the memory enhancement paradigm used in the current study suggest that each of the brain areas (PRh, area V2, and FrC) is not only adequate but also sufficient to induce ORM enhancement and recover ORM, independent of whether there are lesions in the other brain areas. Such organization in the brain might serve as a multilayer of protection against memory loss.

Contrary to brain areas interconnected to ventral pathway circuits, the activation of brain areas that are not interconnected to ventral pathway circuits, such as the parietal cortex, failed to produce any effect on ORM. These findings indicate that the brain areas that directly participate in the ventral pathway circuits can only serve as targets for ORM enhancement. Furthermore, with the use of PRh, area V2, and FrC as examples of brain areas that can be targeted for memory enhancement, we found that RGS14‐mediated ORM enhancement seen after the activation of each of these three brain areas was unaffected by the lesions in other two brain areas, including the PRh area. The finding of lesions in PRh that did not cause any effect on memory enhancement is surprising because it has long been believed that the integrity of PRh is essential for normal ORM function.^4^, ^5^, ^6^ However, our results demonstrate that the PRh is not relevant when ORM enhancement is induced by the increase in synaptic connectivity. Additionally, neurotoxic lesions, as we used in our experiments, do not affect passing nerve fibers^26^, ^27^, ^38^; hence, neural networks passing through the PRh, area V2 and FrC are expected to be unharmed in lesioned animals, and intercommunications among other brain areas are unlikely to be interrupted. As a result, lesions in multiple brain areas might not affect RGS14_414_‐mediated ORM enhancement originated in a nonlesioned brain area. Accordingly, in support of this concept, our results show that ORM enhancement originated in a nonlesioned brain area was not affected even when there was substantial neuronal damage in other two brain areas. These results further emphasize that RGS14‐mediated ORM enhancement as well as recovery rely on ventral pathway circuits and not on interconnected brain areas. Furthermore, occurrence of ORM enhancement even when the PRh is damaged and reversal of ORM in aging rats, a condition in which multiple brain areas are thought to be affected, suggest that activation of a brain area interconnected to ventral pathway circuits might have therapeutic relevance in the recovery of memory in patients or individuals with symptoms of memory dysfunction, and such paradigm could in fact serve as a very effective tool in fight against memory deficits in normal aging and in neurological and neurodegenerative diseases. Moreover, the possibility of being able to choose a brain area that is unharmed or least harmed in an individual patient may be particularly useful for personalized treatment.

In summary, our findings demonstrated that the activation of any one of the brain areas interconnected to ventral pathway circuits is sufficient for ORM enhancement and the reversal of ORM deficit and that the reversal of ORM deficit is dependent on the increase in synaptic connectivity between the local neuronal network and ventral pathway circuits. Additionally, the finding that RGS14_414_ treatment in brain areas that are interconnected to ventral pathway circuits can restore memory deficits might serve as a platform for developing therapeutic strategies for other brain diseases in which neuronal circuits are compromised.

## AUTHOR CONTRIBUTIONS

Z.U.K. developed the overall research concept and the project; M.M., I.N, M.F.L., and Z.U.K. designed the experiments; M.M., I.N., M.F.L., M.E.Q, M.C., and M.F.G performed the experiments; J.F.L., I.J., and C.A.M. assisted with the experiments; and M.M., I.N., M.F.L., and Z.U.K. wrote the manuscript.

## FUNDING INFORMATION

This research was supported by grants from the Ministerio de Ciencia, Innovación y Universidades (PID2022‐136954OB‐I00), Junta de Andalucía (PROYEXCEL‐00422), and Fondo Europeo de Desarrollo Regional (CTS‐586‐G‐FEDER) to Z.U.K.

## CONFLICT OF INTEREST STATEMENT

The authors declare no competing interests.

## Supporting information


Figures S1–S3

## Data Availability

The data that support the findings of this study are available from the corresponding author upon reasonable request.
