# Supplementary material for: Brain areas interconnected to ventral pathway circuits are independently able to induce enhancement in object recognition memory and cause reversal in object recognition memory deficit
Source: CNS Neurosci Ther. 2024 Apr 21;30(4):e14727. doi: 10.1111/cns.14727 (PMC11033489; doi:10.1111/cns.14727)
Supplement: Supplementary file 1 — Figures S1–S3 [file CNS-30-e14727-s001.docx]

Supplemental information for

Brain areas interconnected to ventral pathway circuits are independently able to induce enhancement in object recognition memory and cause reversal in object recognition memory deficit

Mariam Masmudi-Martín, Irene Navarro-Lobato, Manuel F. López-Aranda, María E. Quiros-Ortega, Marta Carretero-Rey, María F. Garcia-Garrido, Juan F. López Téllez, Inmaculada Jiménez-Recuerda, Cristina A. Muñoz de Leon-López, Zafar U. Khan

**Figures (**Figs. S1 to S3)


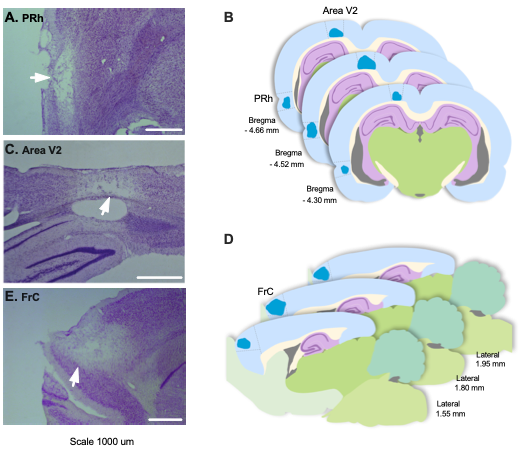


Fig. S1: Lesions in PRh, area V2 and FrC produced significant neuronal damage

rains of rat treated with Ox7-SAP were processed to evaluate the extent of damage in the injection area. (A, C and E) show the images of brain sections after staining with cresyl violet of PRh in A, area V2 in C, and FrC in E. The scale bars shown in image are 1000 µm. Arrow indicates the damage area. (B and D) represent depiction of brain sections after analysis of serial sections from 5 rats, showing maximum expansion of the damage in PRh and area V2 in B and FrC in D (drawings in sky blue color). There was substantial damage in all three brain areas. Bregma of brain sections in B is shown in left side of image and in D is shown in the right side of image.


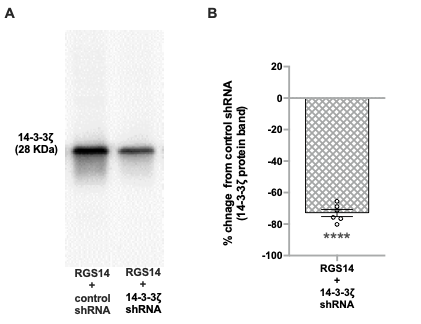


**Fig. S2: Treatment with 14-3-3ζ shRNA reduced the expression of 14-3-3ζ protein**

RGS14-treated rats were subjected to treatment with shRNA of 14-3-3ζ with the goal of knocking down the expression of 14-3-3ζ protein. **(A)** Example Western blots performed with 5 μg of brain homogenate protein showing the expression of 14-3-3ζ protein in control shRNA and 14-3-3ζ-shRNA treated RGS14 rats. (B) The analysis of the optical density values of immunoreactive bands of 14-3-3ζ protein revealed a reduction of 72.98 ± 2.21 % in 14-3-3ζ protein levels. Values in B are presented as the mean ± SEM of 6 experiments (3 experiments from each of the 2 sets of brain homogenate prepared from a pool of 4 animal brain in each set). **** (two-tailed unpaired t test, p < 0.0001).

Fig. S3: RGS14_414_ treatment induced long-lasting ORM enhancer effect

A treatment with RGS14_414_ in PRh, area V2 or FrC caused ORM enhancement, an effect that could be observed even after long time of the treatment. (A) shows the effect of RGS14_414_ treatment in PRh, (B) in area V2, and (C) in FrC. The memory enhancer effect of RGS14_414_ in all three brain areas was seen 3 weeks, 6 week, and 24 weeks after the treatment. In contrast, vehicle treatment produced no such effect. The dotted lines across the figure indicate the threshold at which (0.5 DI and below) the animals were unable to retain object information in memory. **** (Two-way ANOVA with Sidak’s post hoc test, p < 0.0001).

-END-
